# Outcomes after heart transplantation in patients who have undergone a bridge-to-bridge strategy

**DOI:** 10.1016/j.xjon.2022.08.011

**Published:** 2022-09-08

**Authors:** Alice L. Zhou, Eric W. Etchill, Benjamin L. Shou, James J. Whitbread, Iulia Barbur, Katherine A. Giuliano, Ahmet Kilic

**Affiliations:** a Johns Hopkins University School of Medicine, Baltimore, Md; b Division of Cardiac Surgery, Department of Surgery, Johns Hopkins University School of Medicine, Baltimore, Md

**Keywords:** mechanical circulatory support, heart transplant, intra-aortic balloon pump, extracorporeal membrane oxygenation, ventricular assist devices, transplant outcomes, BTB, bridge-to-bridge, BTT, bridge-to-transplant, CO, cardiac output, ECMO, extracorporeal membrane oxygenation, IABP, intra-aortic balloon pump, LVAD, left ventricular assist device, MCS, mechanical circulatory support, mPAP, mean pulmonary arterial pressure, OPTN, Organ Procurement and Transplantation Network, PA, pulmonary artery, PCWP, pulmonary capillary wedge pressure, tVAD, temporary ventricular assist device, TAH, total artificial heart, UNOS, United Network for Organ Sharing

## Abstract

**Objectives:**

We compared posttransplant outcomes between patients bridged from temporary mechanical circulatory support to durable left ventricular assist device before transplant (bridge-to-bridge [BTB] strategy) and patients bridged from temporary mechanical circulatory support directly to transplant (bridge-to-transplant [BTT] strategy).

**Methods:**

We identified adult heart transplant recipients in the Organ Procurement and Transplantation Network database between 2005 and 2020 who were supported with extracorporeal membrane oxygenation, intra-aortic balloon pump, or temporary ventricular assist device as a BTB or BTT strategy. Kaplan-Meier survival analysis and Cox regressions were used to assess 1-year, 5-year, and 10-year survival. Posttransplant length of stay and complications were compared as secondary outcomes.

**Results:**

In total, 201 extracorporeal membrane oxygenation (61 BTB, 140 BTT), 1385 intra-aortic balloon pump (460 BTB, 925 BTT), and 234 temporary ventricular assist device (75 BTB, 159 BTT) patients were identified. For patients supported with extracorporeal membrane oxygenation, intra-aortic balloon pump, or temporary ventricular assist device, there were no differences in survival between BTB and BTT at 1 and 5 years posttransplant, as well as 10 years posttransplant even after adjusting for baseline characteristics. The extracorporeal membrane oxygenation BTB group had greater rates of acute rejection (32.8% vs 13.6%; *P* = .002) and lower rates of dialysis (1.6% vs 21.4%; *P* < .001). For intra-aortic balloon pump and temporary ventricular assist device patients, there were no differences in posttransplant length of stay, acute rejection, airway compromise, stroke, dialysis, or pacemaker insertion between BTB and BTT recipients.

**Conclusions:**

BTB patients have similar short- and midterm posttransplant survival as BTT patients. Future studies should continue to investigate the tradeoff between prolonged temporary mechanical circulatory support versus transitioning to durable mechanical circulatory support.

## Graphical abstract

Graphical abstract summarizing results of the study.

**Figure:**
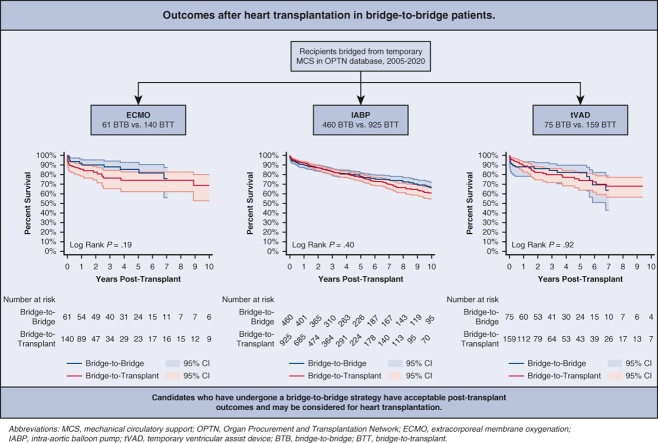

**Figure:**
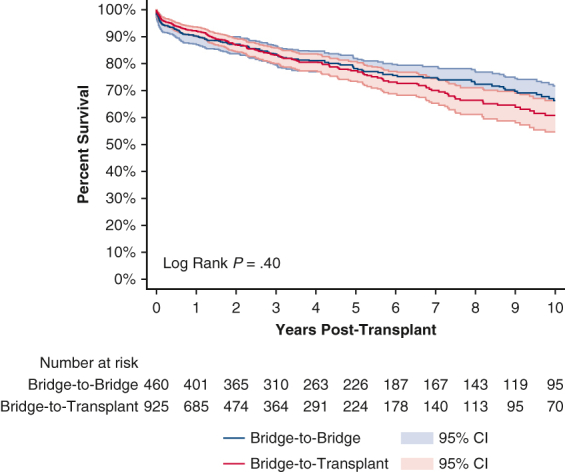
Ten-year survival curve for IABP bridge-to-bridge and bridge-to-transplant patients.

**Central Message**

Patients who have undergone a bridge-to-bridge strategy have similar posttransplant outcomes as patients undergoing a bridge-to-transplant strategy and may be considered for transplantation.

**Perspective**

Given the recent increase in temporary MCS as a bridge to definitive therapy in patients with heart failure, outcomes of different bridging strategies from temporary MCS must be understood. Our study demonstrates that patients who have undergone a bridge-to-bridge strategy have acceptable posttransplant outcomes and may be considered for transplantation.

Temporary mechanical circulatory support (MCS) devices are increasingly being utilized as a bridge to definitive therapy in patients with end-stage heart failure.^1^ Following the implementation of the 2018 United Network for Organ Sharing (UNOS) heart allocation policy,^2^ overall temporary MCS utilization in advanced heart failure patients has increased in US transplant centers.^3^ Additionally, the percentage of patients bridged directly from temporary MCS to transplantation has increased from 13.5% before the policy change to 44.5% shortly following the policy change, with the greatest increase observed in patients bridged from intra-aortic balloon pumps (IABP) and extracorporeal membrane oxygenation (ECMO).^4^

Due to these recent shifts in practice, it is important to understand the outcomes of patients supported with temporary MCS devices. These patients may be bridged to a durable MCS device before transplantation (bridge-to-bridge [BTB] strategy) or directly to transplantation (bridge-to-transplant [BTT] strategy). Although it has been shown that BTT from temporary MCS leads to worse 1-year posttransplant outcomes than BTT from durable MCS,^5^ there have been limited studies examining posttransplant outcomes of BTB patients. A previous study published in 2013 found that patients who were bridged from ECMO to a left ventricular assist device (LVAD) before transplantation had improved 1-year and 5-year posttransplant survival compared with those bridged directly from ECMO to transplantation.^6^ However, BTB strategies from IABPs and temporary VADs (tVADs) have not been studied, and a more contemporary cohort of ECMO BTB patients has not been investigated.

This study sought to address the gap in knowledge on posttransplant outcomes associated with a BTB strategy before transplantation. We conducted a national retrospective analysis of posttransplant outcomes in BTB patients using the Organ Procurement and Transplantation Network (OPTN) database. We also hypothesized that patients undergoing a BTB strategy would have similar posttransplant survival as those undergoing a BTT strategy.

## Materials and Methods

### Data Source

This national retrospective cohort study was conducted using the OPTN registry with follow-up until March 2021. The OPTN database contains information on all transplant candidates, recipients, and donors, as well as waitlist and posttransplant outcomes in the United States. This study was approved by the Johns Hopkins University Institutional Review Board (IRB00159748) on January 5, 2018.

### Waitlist Analysis

**Figure 1:**
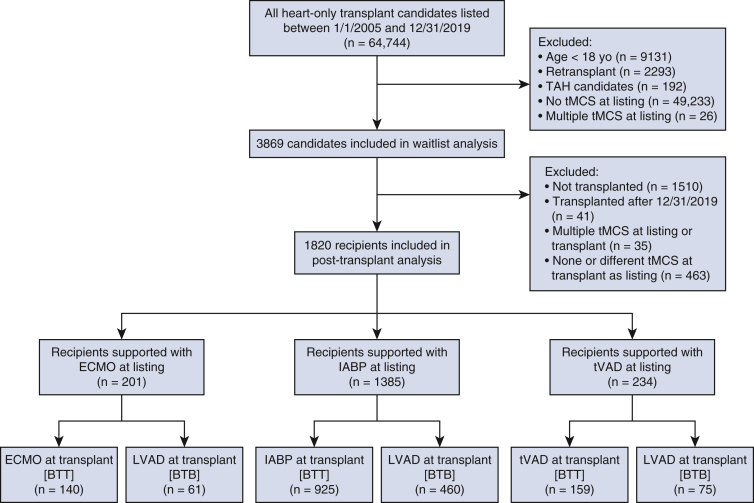
Flow diagram showing inclusion and exclusion criteria to generate study population. *TAH*, Total artificial heart; *MCS*, mechanical circulatory support; *ECMO*, extracorporeal membrane oxygenation; *IABP*, intra-aortic balloon pump; *tVAD*, temporary ventricular assist device; *LVAD*, left ventricular assist device; *BTT*, bridge-to-transplant; *BTB*, bridge-to-bridge.

The OPTN database was retrospectively reviewed to identify all adult (aged ≥18 years) heart transplant candidates listed with a temporary MCS device between January 1, 2005, and December 31, 2019 (Figure 1). Multiorgan, retransplant, and total artificial heart candidates were excluded. Due to clinical heterogeneity and differences in device indication, temporary MCS was subdivided into ECMO, IABP, and tVAD. Fine-Gray competing-risk regressions^7^ were used to construct risk-adjusted rates of waitlist transplant, waitlist death, and LVAD implantation within 1 year since listing for candidates supported with temporary MCS at listing. In these models, LVAD implantation was considered a competing event, and candidates were censored at the time of implant, regardless of ultimate waitlist outcome.

### BTB and BTT Groups

The remaining analyses investigated posttransplant outcomes in BTB versus BTT recipients. The study population described above was further refined to include only candidates who received a heart transplant before December 31, 2019, to allow for sufficient posttransplant follow-up time (Figure 1). Recipients were categorized as BTB or BTT based on their pretransplant bridging strategy. BTB recipients were defined as those supported with temporary MCS at listing and durable LVAD at transplant. BTT recipients served as a control group and were defined as those supported with the same temporary MCS at both listing and transplant. Recipients supported with both IABP and tVAD at either listing or transplant were excluded. Recipients supported with ECMO and either IABP or tVAD were considered as patients receiving ECMO requiring left ventricle unloading and were therefore included in the study as an patient receiving ECMO.

### Baseline Characteristics

Normality of all variables were assessed using Shapiro-Wilk testing and histogram visualization. Baseline characteristics of patients receiving ECMO, IABP, and tVAD were compared using 1-way analysis of variance for parametric continuous variables, Kruskal-Wallis tests for nonparametric continuous variables, and χ^2^ or Fisher exact (if n ≤ 5 for any group) tests for categorical variables. Within each temporary MCS group, baseline characteristics were compared between patients undergoing the BTB and BTT strategies using Student *t* tests for parametric continuous variables, Wilcoxon rank-sum tests for nonparametric continuous variables, and χ^2^ or Fisher exact (if n ≤ 5 for any group) for categorical variables. Parametric continuous, nonparametric continuous, and categorical variables were reported as mean ± SD, median (interquartile range [IQR]), and number (percent), respectively.

### Survival Analyses

The primary outcomes were 1-year, 5-year, and 10-year posttransplant survival. Kaplan-Meier analyses and univariate Cox proportional hazards regressions were used to investigate survival at each of the time points. Additionally, 5-year and 10-year conditional survival analyses were performed given the dynamic risk of mortality after heart transplant noted in previously published studies.^8^ Five-year conditional survival was calculated by restricting the risk set to recipients who survived to 1-year posttransplant. Similarly, 10-year conditional survival was calculated by restricting the risk set to those who survived to 5 years posttransplant.

Multivariable Cox proportional hazards regressions were used to investigate 1-year and 10-year unconditional survival. Covariates were identified a priori based on previous UNOS reports^9^ and clinical judgment and included recipient age, recipient sex, recipient ethnicity, recipient body mass index, donor age, donor sex, donor ethnicity, graft ischemic time, transplant year, and LVAD type.

### Secondary Outcomes

Secondary outcomes were posttransplant length of stay, acute rejection, acute rejection requiring treatment, stroke, new-onset dialysis, and pacemaker insertion. Normality of length of stay was assessed using Shapiro-Wilk testing and histogram visualization and was found to be nonparametric. Length of stay was reported as median (IQR) and assessed using Wilcoxon rank-sum tests. Other secondary outcomes were reported as number (%) and were assessed using χ^2^ or Fisher exact tests (if n ≤ 5 for any group) tests.

### Subgroup Analysis

A subgroup analysis of patients from the most recent quartile of the study period (2017-2020) was performed. Given the short follow-up time, 30-day and 1-year unconditional mortality was compared using log-rank tests. Secondary outcomes were assessed as described above. All statistical analyses were performed using Stata version 15.1 (StataCorp).

## Results

### Waitlist Analysis

**Figure E1:**
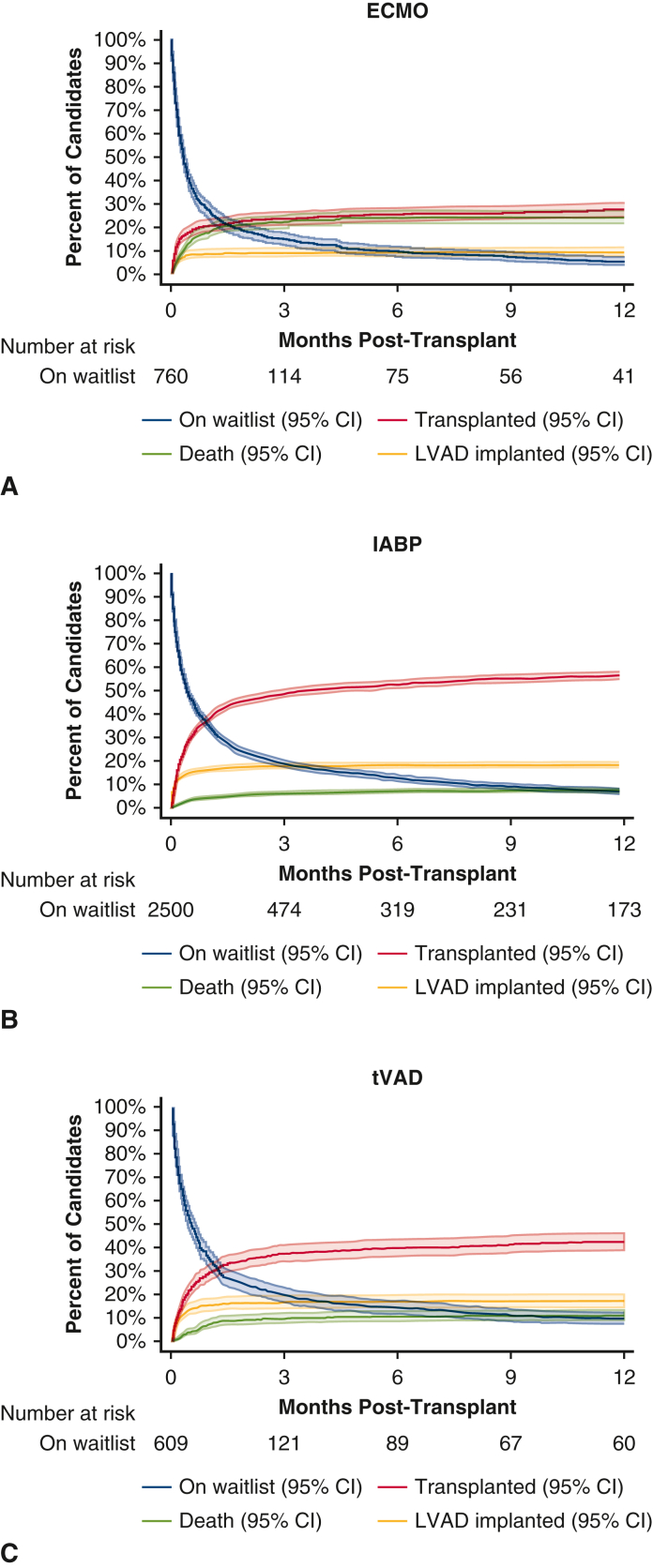
Waitlist outcomes for candidates initially listed with (A) extracorporeal membrane oxygenation (*ECMO*), (B) intra-aortic balloon pump (*IABP*), and (C) temporary ventricular assist device (*tVAD*). 95% confidence intervals (CI) in shaded area.

A total of 3869 waitlist candidates were included in the study, of whom 760 were supported with ECMO at listing, 2500 with IABP at listing, and 609 with tVAD at listing. Of the 760 ECMO candidates, 27.4% were transplanted, 9.5% received an LVAD implant, 3.9% received a non-LVAD device, 24.2% died on the waitlist, and 29.7% were delisted for other reasons within the first year after listing (Figure E1). Of the 2500 IABP candidates, 56.6% were transplanted, 18.3% received an LVAD implant, 3.4% received a non-LVAD device, 7.6% died on the waitlist, and 7.2% were delisted for other reasons within the first year after listing. Of the 609 tVAD candidates, 42.5% were transplanted, 17.2% received an LVAD implant, 6.7% received a non-LVAD device, 11.2% died on the waitlist, and 12.5% were delisted for other reasons within the first year after listing.

### Study Population

A total of 1820 patients undergoing temporary MCS BTB and BTT strategies met the inclusion criteria (Figure 1). Of these, 201 were bridged from ECMO (61 BTB, 140 BTT), 1385 from IABP (460 BTB, 925 BTT), and 234 from tVAD (75 BTB, 159 BTT). Patients supported with ECMO at listing were on average younger at transplant (*P* < .0001) and more predominantly white (*P* < .0001) compared with patients supported with IABP or tVAD (Table 1). Patients bridged from ECMO also had a shorter median waitlist time of 7 days (IQR, 3-55 days), compared with 20 days (IQR, 6-108 days) for IABP patients and 25 days (IQR, 6-144 days) for tVAD patients (*P* < .001). At transplant, patients bridged from IABP had significantly higher pulmonary capillary wedge pressure (PCWP) (*P* = .007) and mean pulmonary artery (PA) pressure (*P* < .0001) than patients bridged from ECMO or tVAD.

**Table 1:** Baseline characteristics of study population by temporary mechanical circulatory support device

Variable	ECMO (n = 201)	IABP (n = 1385)	tVAD (n = 234)	*P* value
Age at transplant (y)	46 (33-57)	57 (48-63)	53 (42-60)	**<.001**
Male sex	141 (70.1)	1040 (75.1)	177 (75.6)	.30
Ethnicity				**<.001**
White	155 (77.1)	864 (62.4)	147 (62.8)	
Black	23 (11.4)	328 (23.7)	41 (17.5)	
Hispanic	8 (4.0)	121 (8.7)	33 (14.1)	
Other	15 (7.5)	78 (5.6)	13 (5.6)	
Total days on waitlist	7 (3-55)	20 (6-108)	25 (6-144)	**<.001**
Hemodynamic status at listing				
Cardiac output (L/min)	3.9 (3.0-5.1)	3.8 (3.0-4.7)	3.8 (3.1-5.3)	.29
PCWP (mm Hg)	25 (18-31)	24 (17-30)	24 (18-30)	.19
mPAP (mm Hg)	30 (23-38)	33 (27-40)	32 (23-38)	**<.001**
PA systolic pressure (mm Hg)	42 (31-53)	48 (39-57)	44 (34-51)	**<.001**
PA diastolic pressure (mm Hg)	24 (17-32)	25 (19-30)	24 (17-29)	.22
Hemodynamic status at transplant				
Cardiac output (L/min)	4.4 (3.2-5.7)	4.1 (3.3-5.1)	4.2 (3.3-5.8)	.058
PCWP (mm Hg)	18 (11-27)	21 (14-28)	18 (13-27)	**.007**
mPAP (mm Hg)	25 (20-34)	30 (23-38)	27 (20-34)	**<.001**
PA systolic pressure (mm Hg)	36 (30-47)	44 (34-55)	39 (29-49)	**<.001**
PA diastolic pressure (mm Hg)	19 (12-25)	22 (16-28)	20 (14-26)	**<.001**

Values are presented as median (interquartile range) or n (%). Statistical significant (*P* < .05) denoted with boldface type. *ECMO*, Extracorporeal membrane oxygenation; *IABP*, intra-aortic balloon pump; *tVAD*, temporary ventricular assist device; *PCWP*, pulmonary capillary wedge pressure; *mPAP*, mean pulmonary arterial pressure; *PA*, pulmonary artery.

### Baseline Characteristics and Outcomes of Patients Bridged from ECMO

Of the patients bridged from ECMO, patients undergoing a BTB strategy had higher cardiac output (CO) (4.7 [4.0-5.8] vs 3.8 [3.0-5.6] L/min; *P* = .007) and lower PCWP (15 [11-22] vs 20 [13-28] mm Hg; *P* = .013); values are presented as median (interquartile range) (Table 2). The patients undergoing ECMO BTB strategy spent significantly longer on the waitlist (191 [76-319] vs 4 [2-8] days; *P* < .001) and had greater follow-up time (4.0 [2.5-6.0] vs 1.0 [1.0-3.0] years; *P* < .001) relative to the patients undergoing the ECMO BTT strategy.

**Table 2:** Baseline characteristics at listing and at transplant of bridge-to-bridge and bridge-to-transplant recipients

Variable	ECMO			IABP			tVAD		
	BTB (n = 61)	BTT (n = 140)	*P* value	BTB (n = 460)	BTT (n = 925)	*P* value	BTB (n = 75)	BTT (n = 159)	*P* value
Age at transplant (y)	46 (34-58)	47 (31-56)	.41	56 (47-62)	57 (48-63)	.12	54 (46-60)	53 (39-61)	.45
Male sex	42 (68.9)	99 (70.7)	.79	361 (78.5)	679 (73.4)	**.040**	59 (78.7)	118 (74.2)	.46
Ethnicity			**.044**			.99			.19
White	46 (75.4)	109 (77.9)		286 (62.2)	578 (62.5)		45 (60.0)	102 (64.2)	
Black	12 (19.7)	11 (7.9)		108 (23.5)	220 (23.8)		18 (24.0)	23 (14.5)	
Hispanic	1 (1.6)	7 (5.0)		41 (8.9)	80 (8.6)		7 (9.3)	26 (16.4)	
Other	2 (3.3)	13 (9.3)		25 (5.4)	47 (5.1)		5 (6.7)	8 (5.0)	
BMI	26.1 (23.5-30.3)	26.4 (23.3-30.2)	.93	26.6 (23.6-30.2)	25.8 (23.0-29.3)	**.003**	27.0 (23.9-30.8)	26.6 (23.7-29.7)	.82
Diabetes	13 (21.3)	22 (15.7)	.34	140 (30.4)	249 (26.9)	.17	17 (22.7%)	39 (24.5)	.76
Hemodynamic status at listing									
Cardiac output (L/min)	4.1 (3.3-4.8)	3.7 (3.0-5.1)	.41	3.9 (3.0-4.9)	3.8 (3.1-4.7)	.49	3.8 (3.2-5.5)	3.8 (3.1-5.3)	.79
PCWP (mm Hg)	25 (17-32)	25 (18-31)	.85	25 (18-30)	23 (17-29)	**.027**	25 (17-30)	24 (18-30)	.72
mPAP (mm Hg)	32 (24-38)	30 (22-38.5)	.49	35 (29-41)	33 (26-39)	**<.001**	33 (27-37)	30 (23-38)	.64
PA systolic pressure (mm Hg)	43 (31-50)	42 (31-53)	.87	50 (42-58)	48 (38-55)	**<.001**	47 (36-51)	42 (34-52)	.52
PA diastolic pressure (mm Hg)	25 (20-30)	23 (17-32)	.49	25 (21-30)	24 (19-30)	**<.001**	23 (18-29)	24 (17-30)	.92
Ischemic time (h)	3.3 (2.6-3.9)	3.3 (2.7-3.9)	.78	3.3 (2.5-3.8)	3.2 (2.6-3.8)	.76	3.2 (2.7-3.9)	3.4 (2.7-4.1)	.13
Total days on waitlist	191 (76-319)	4 (2-8)	**<.001**	185 (101-343)	10 (4-22)	**<.001**	266 (104-415)	12 (4-32)	**<.001**
Follow-up time (y)	4.0 (2.5-6.0)	1.0 (1.0-3.0)	**<.001**	5.0 (2.3-9.1)	2.0 (1.0-5.0)	**<.001**	3.7 (1.2-5.4)	2.0 (1.0-6.0)	.14
Transplant year			**<.001**			**<.001**			.097
2005-2008	7 (11.5)	14 (10.0)		123 (26.7)	115 (12.4)		3 (4.0)	3 (1.9)	
2009-2012	8 (13.1)	10 (7.1)		129 (28.0)	106 (11.5)		15 (20.0)	34 (21.4)	
2013-2016	29 (47.5)	23 (16.4)		123 (26.7)	208 (22.5)		33 (44.0)	48 (30.2)	
2017-2020	17 (27.9)	93 (66.4)		85 (18.5)	496 (53.6)		24 (32.0)	74 (46.5)	
Hemodynamic status at transplant									
Cardiac output (L/min)	4.7 (4.0-5.8)	3.8 (3.0-5.6)	**.007**	4.5 (3.6-5.4)	3.8 (3.1-4.9)	**<.001**	4.2 (3.6-5.5)	4.1 (3.2-5.9)	.65
PCWP (mm Hg)	15 (11-22)	20 (13-28)	**.013**	17 (11-25)	22 (16-28)	**<.001**	16 (11-21)	20 (15-28)	**.001**
mPAP (mm Hg)	23 (18-31)	27 (20-36)	.057	27 (21-36)	32 (25-39)	**<.001**	25 (19-32)	30 (22-36)	**.005**
PA systolic pressure (mm Hg)	36 (27-45)	37 (31-48)	.18	40 (30-52)	46 (36-55)	**<.001**	35 (29-45)	40 (31-50)	**.042**
PA diastolic pressure (mm Hg)	16 (10-23)	20 (13-27)	**.05**	19 (13-27)	23 (18-29)	**<.001**	16 (14-22)	21 (15-27)	**.004**
Donor age (y)	34 ± 12	33 ± 11	.30	32 ± 12	32 ± 11	.88	31 ± 11	32 ± 11	.24
Donor male sex	39 (63.9)	96 (68.6)	.52	335 (72.8)	642 (69.4)	.19	53 (70.7)	119 (74.8)	.50
Donor ethnicity			.21			.28			.85
White	41 (67.2)	103 (73.6)		307 (66.7)	592 (64.0)		49 (65.3)	98 (61.6)	
Black	13 (21.3)	18 (12.9)		73 (15.9)	169 (18.3)		8 (10.7)	23 (14.5)	
Hispanic	7 (11.5)	14 (10.0)		66 (14.3)	147 (15.9)		15 (20.0)	33 (20.8)	
Other	0 (0.0)	5 (3.6)		14 (3.0)	17 (1.8)		3 (4.0)	5 (3.1)	
Donor BMI	25.5 (22.4-27.8)	26.0 (23.2-30.3)	.13	26.8 (23.5-30.4)	26.1 (23.2-30.2)	.21	26.0 (23.3-29.6)	26.6 (23.1-31.2)	.44

Values are presented as median (interquartile range), n (%), or mean ± SD. Statistical significant (*P* < .05) denoted with boldface type. *ECMO*, Extracorporeal membrane oxygenation; *IABP*, intra-aortic balloon pump; *tVAD*, temporary ventricular assist device; *BTB*, bridge to bridge; *BTT*, bridge to transplant; *BMI*, body mass index; *PCWP*, pulmonary capillary wedge pressure; *mPAP*, mean pulmonary arterial pressure; *PA*, pulmonary artery.

**Figure 2:**
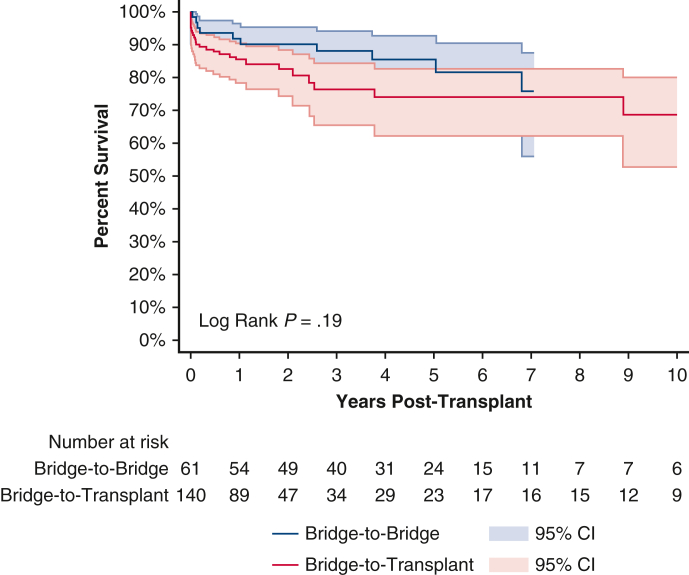
Ten-year posttransplant survival of bridge-to-bridge versus bridge-to-transplant from extracorporeal membrane oxygenation. Individual plots were truncated when the number at risk fell below 10. 95% confidence intervals (CI) in shaded area.

Posttransplant survival rate for patients undergoing ECMO BTB strategy was 91.8%, 85.5%, and 75.8% at 1 year, 5 years, and 10 years, respectively (Figure 2 and Table E1). For patients undergoing an ECMO BTB strategy who lived to 1 year posttransplant, 5-year survival was 93.1%, and for those who lived to 5 years, 10-year survival was 88.6%. For patients undergoing an ECMO BTT strategy, survival rate was 85.5%, 73.9%, and 68.7% at 1 year, 5 years, and 10 years, respectively. Five-year conditional survival was 86.5% and 10-year conditional survival was 92.9%.

On univariate analysis, patients undergoing an ECMO BTB strategy compared with patients undergoing an ECMO BTT strategy had similar 1-year (*P* = .21), 5-year (*P* = .11), or 10-year (*P* = .19) unconditional survival. On multivariable analysis, patients undergoing an ECMO BTB strategy still had similar 1-year mortality (adjusted hazard ratio [aHR], 0.75; 95% CI, 0.05-10.45; *P* = .83) (Table E2) and 10-year mortality (aHR, 0.38; 95% CI, 0.07-2.04; *P* = .26) (Table E3) compared with patients undergoing an ECMO BTT strategy. Patients undergoing ECMO BTB and BTT strategies had similar postoperative lengths of stay (20 days [IQR, 13-29 days] vs 21 days [IQR, 15-38 days]; *P* = .11) (Table 3). However, patients undergoing an ECMO BTB strategy had significantly greater rates of predischarge acute rejection (32.8% vs 13.6%; *P* = .002) and significantly lower rates of predischarge dialysis (1.6% vs 21.4%; *P* < .001) compared with patients undergoing an ECMO BTT strategy (Table 3).

**Table 3:** Postoperative outcomes in bridge-to-bridge (BTB) versus bridge-to-transplant (BTT) patients

MCS device	Postoperative outcomes	BTB	BTT	*P* value
ECMO		n = 61	n = 160	
	Length of stay (d)	20 (13-29)	21 (15-38)	.11
	Acute rejection	20 (32.8)	19 (13.6)	**.002**
	Treated acute rejection	7 (11.5)	8 (5.7)	.15
	Airway compromise	0 (0.0)	0 (0.0)	
	Stroke	1 (1.6)	14 (10.0)	.042
	Dialysis	1 (1.6)	30 (21.4)	**<.001**
	Pacemaker	0 (0.0)	0 (0.0)	
IABP		n = 460	n = 925	
	Length of stay (d)	16 (11-23)	16 (11-23)	1.00
	Acute rejection	117 (25.4)	214 (23.1)	.34
	Treated acute rejection	52 (11.3)	84 (9.1)	.19
	Airway compromise	3 (0.7)	2 (0.2)	.34
	Stroke	13 (2.8)	25 (2.7)	.89
	Dialysis	62 (13.5)	115 (12.4)	.58
	Pacemaker	12 (2.6)	31 (3.4)	.45
tVAD		n = 75	n = 159	
	Length of stay (d)	16 (12-30)	19 (13-29)	.23
	Acute rejection	17 (22.7)	42 (26.4)	.54
	Treated acute rejection	11 (14.7)	23 (14.5)	.97
	Airway compromise	0 (0)	0 (0)	
	Stroke	5 (6.7)	8 (5.0)	.61
	Dialysis	8 (10.7)	20 (12.6)	.67
	Pacemaker	3 (4.0)	3 (1.9)	.39

Values are presented as median (interquartile range) or n (%). Statistical significant (*P* < .05) denoted with boldface type. *MCS*, Mechanical circulatory support; *ECMO*, extracorporeal membrane oxygenation; *IABP*, intra-aortic balloon pump; *tVAD*, temporary ventricular assist device.

### Baseline Characteristics and Outcomes of Patients Bridged from IABP

Within the IABP group, patients undergoing a BTB strategy were younger at listing (55 years [IQR, 46-61 years] vs 57 years [IQR, 48-63 years]; *P* = .005), more predominantly men (78.5% vs 73.4%; *P* = .04), and had greater body mass index (26.6 [IQR, 23.6-30.2] vs 25.8 [23.0-29.3]; *P* = .003) relative to the patients undergoing the BTT strategy (Table 2). Patients undergoing the IABP BTB strategy also had significantly higher PCWP (*P* < .027) and mean.

29 pulmonary arterial pressure (mPAP) (*P* < .001) at listing than the patients undergoing an IABP BTT strategy. At transplant, patients undergoing an IABP BTB strategy had significantly higher CO (*P* < .001), lower PCWP (*P* < .001), and lower mPAP (*P* < .001) than the patients undergoing an IABP BTT strategy (Table 2). Patients undergoing an IABP BTB strategy also had significantly more days spent on the waitlist (185 days [IQR, 101-343 days] vs 10 days [4-22 days]; *P* < .001) and longer follow-up time (5.0 [IQR, 2.3-9.1 years] vs 2.0 years [1.0-5.0 years]; *P* < .001).

**Figure 3:**
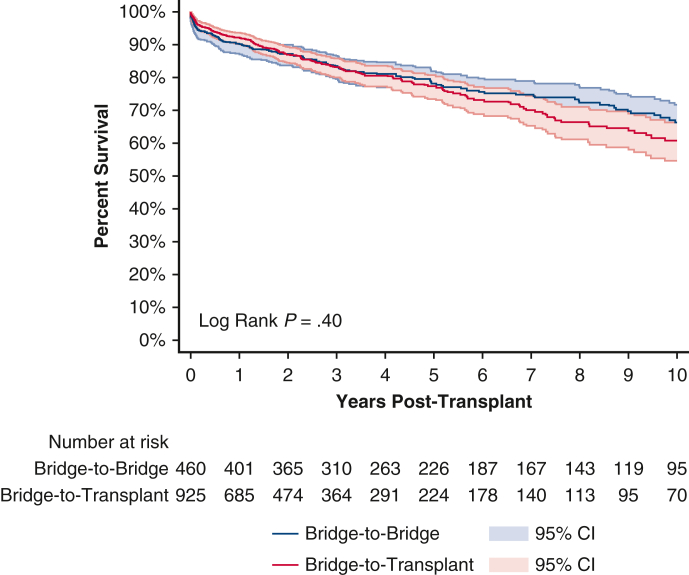
Ten-year posttransplant survival of bridge-to-bridge versus bridge-to-transplant from intra-aortic balloon pump. Individual plots were truncated when the number at risk fell below 10. 95% confidence intervals (CI) in shaded area.

Posttransplant survival rate for patients undergoing an IABP BTB and BTT strategy were 90.1% and 92.2% at 1 year; 78.2% and 77.3% at 5 years; and 66.3% and 60.8% at 10 years, respectively (Figure 3 and Table E1). Conditional 5-year survival was 86.8% for patients undergoing an IABP BTB strategy and 83.9% for patients undergoing an IABP BTT strategy. Conditional 10-year survival was 84.9% for patients undergoing an IABP BTB strategy and 78.7% for patients undergoing an IABP BTT strategy.

For patients bridged from an IABP, there were no significant differences on univariate analysis between the BTB and BTT groups for 1-year (*P* = .20), 5-year (*P* = .96), or 10-year (*P* = .40) unconditional survival. After adjusting for baseline characteristics, 1-year unconditional (aHR, 1.32; 95% CI, 0.70-2.49; *P* = .39) (Table E2) and 10-year unconditional survival (aHR, 0.82; 95% CI, 0.64-1.05; *P* = .12) (Table E3) was still similar between patients undergoing IABP BTB and BTT strategies. There was no significant difference between the BTB and BTT groups with regard to postoperative lengths of stay, acute rejection, airway compromise, stroke, dialysis, or pacemaker insertion (Table 3).

### Outcomes of Patients Bridged from tVAD

Compared with the tVAD BTT group, the tVAD BTB group had similar age, sex, ethnicity, BMI, rates of diabetes, and hemodynamics at listing (Table 2). At transplant, the tVAD BTB group had lower PCWP (*P* = .001) and mPAP (*P* = .005) than the tVAD BTT group, with no difference in CO (*P* = .65). The BTB group also spent significantly longer on the waitlist (266 days [IQR, 104-415 days] vs 12 [IQR, 4-32 days]; *P* < .001).

**Figure 4:**
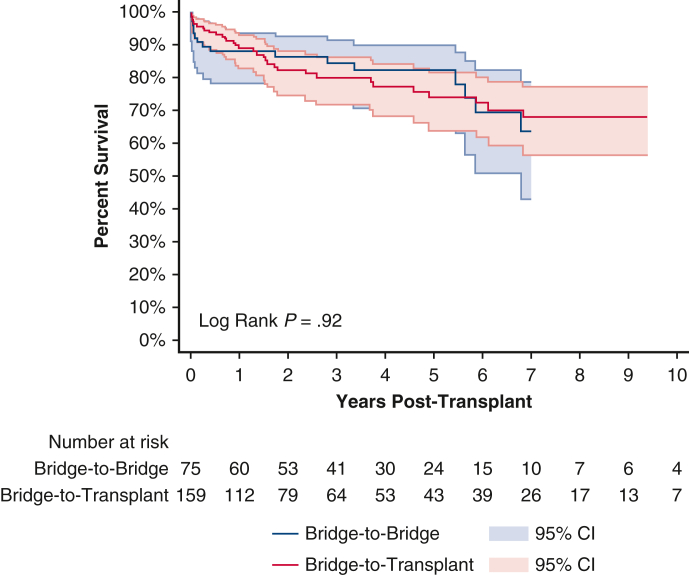
Ten-year posttransplant survival of bridge-to-bridge versus bridge-to-transplant from temporary ventricular assist device. Individual plots were truncated when the number at risk fell below 10. 95% confidence intervals (CI) in shaded area.

Posttransplant survival rate for patients undergoing an tVAD BTB strategy was 88.0%, 82.4%, and 55.7% at 1 year, 5 years, and 10 years, respectively (Figure 4 and Table E1). For patients undergoing an tVAD BTB strategy who lived to 1 year posttransplant, 5-year survival was 93.6%, and for those who lived to 5 years, 10-year survival was 55.7%. For patients undergoing an tVAD BTT strategy, survival rate was 89.0%, 73.9%, and 68.0% at 1 year, 5 years, and 10 years respectively. Five-year conditional survival was 83.1% and 10-year conditional survival was 91.9%.

Of patients bridged from a tVAD, the BTB and BTT groups had no significant difference in 1-year (*P* = .75), 5 year (*P* = .43), or 10 year (*P* = .92) unconditional survival. On multivariable analysis, there were no differences in 1-year (aHR, 1.17; 95% CI, 0.25-5.60; *P* = .84) (Table E2) or 10 year (aHR, 1.33; 95% CI, 0.27-6.60; *P* = .80) (Table E3) unconditional survival. The BTB group had a similar length of stay as the BTT group (16 days [IQR, 12-30 days] vs 19 days [13-29 days]; *P* = .23) (Table 3). Both groups were found to have similar rates of predischarge acute rejection (*P* = .54), stroke (*P* = .61), dialysis (*P* = .67), and pacemaker insertion (*P* = .39).

### Subgroup Analysis of 2017-2020 Transplants

For patients who underwent transplant during the most recent quartile of the study period (2017-2020), patients undergoing an IABP BTB strategy had greater rates of acute rejection than patients undergoing an IABP BTT strategy (34.1% vs 21.0%; *P* = .008) and patients undergoing an tVAD BTB strategy had greater rates of stroke than patients undergoing an tVAD BTT strategy (8.0% vs 0.0%; *P* = .01). There were no statistically significant differences in 30-day or 1-year mortality between BTB and BTT transplants (Table E4).

## Discussion

Although temporary MCS use for the management of patients with heart failure has increased during recent years,^3^^,^^4^ little is known about posttransplant outcomes in patients bridged from a temporary MCS device to a durable LVAD before heart transplantation.^10^ The results from our waitlist analysis demonstrated that 9.5% of EMCO candidates, 18.3% of IABP candidates, and 17.2% of tVAD candidates underwent LVAD implantation during the first year of being on the waitlist. Given these findings, we conducted a retrospective review of the United States experience with transplantation in recipients who have undergone a BTB strategy. The results from this study suggest that patients undergoing a BTB strategy have similar posttransplant survival as patients undergoing an temporary MCS BTT strategy but longer waitlist times.

Significant differences between the temporary MCS populations were observed. Patients in the ECMO group were younger than patients in the IABP and tVAD groups, and patients in the IABP group were older than ECMO and tVAD group patients, consistent with previous studies.^11^ Patients in the IABP group had higher PCWP at transplant and higher PA pressures at both listing and transplant than ECMO and tVAD group patients. This finding is consistent with previous data that found greater PCWP in IABP group patients relative to tVAD patients and may reflect the increased hemodynamic support provided by ECMO and tVADs compared with IABPs.^12^^,^^13^ When comparing baseline characteristics between patients undergoing BTB and BTT strategies, PCWP and PA pressures at transplant were significantly lower in the BTB strategy patients compared with BTT strategy patients for both the IABP and tVAD groups, suggesting that transitioning patients to a durable MCS device could provide hemodynamic stabilization. Additionally, for the ECMO and IABP groups, significantly greater proportions of patients undergoing the BTT strategy were transplanted between 2017 and 2020, a finding consistent with data showing an increase in direct BTT from temporary MCS following the 2018 UNOS allocation policy revision.^3^^,^^4^ For all groups, patients undergoing a BTB strategy spent significantly longer on the waitlist than patients undergoing a BTT strategy, which is expected given that patients supported with temporary MCS receive higher priority status under the new UNOS allocation policy^2^ and that patients with durable LVADs tend to be in a more stable condition.

This study was the first to investigate posttransplant outcomes in patients undergoing a BTB strategy initially supported with IABP or tVAD, and we demonstrated no differences in 1-, 5-, and 10-year unconditional posttransplant survival in these patients compared with BTT recipients. For the ECMO group, our results also demonstrated similar 1- and 5-year survival, as well as 10-year adjusted survival in patients undergoing a BTB strategy compared with patients undergoing a BTT strategy. Our short-term outcomes are in contrast with the results of a study conducted by Karamlou and colleagues,^6^ who showed that patients undergoing an ECMO-to-LVAD strategy had better 1-year and 5-year posttransplant survival relative to patients undergoing an ECMO BTT strategy. Notably, we found a 1-year survival rate of 85% in our ECMO BTT population, compared with the 62% survival rate reported by Karamlou and colleagues.^6^ It is possible that these differences reflect characteristics of the study population. The analysis by Karamlou and colleagues^6^ included both adult and pediatric patients transplanted between 2000 and 2010. Our population (adult recipients between 2005 and 2020) represents a more contemporary cohort of patients who had greater survival rates in the ECMO BTT population. Additionally, our subgroup analysis of ECMO BTB and BTT transplants from 2017 to 2020 showed no difference in 30-day or 1-year mortality. Improved survival in ECMO BTT patients over time could explain why posttransplant outcomes were similar in our ECMO BTT and ECMO BTB populations. On the whole, our results on posttransplant survival in patients undergoing a BTB strategy was comparable to previously reported survival data for the general heart transplant population^8^ and for recipients bridged with only a durable LVAD,^9^ suggesting that support with multiple devices and undergoing additional procedures pretransplant did not significantly influence survival posttransplant.

When looking at postoperative complications, the ECMO BTB group was shown to have significantly greater episodes of acute rejection, but no differences in episodes of treated acute rejection. Similar findings were demonstrated in the IABP subgroup analysis of 2017-2020 transplants. This is consistent with previous studies showing increased allosensitization in VAD patients^14^^,^^15^ without an increase in clinically relevant rejection episodes.^16^^,^^17^ Reassuringly, for both of these groups, the greater rates of acute rejection did not translate to differences in short-term mortality. Additionally, patients undergoing an ECMO BTB strategy were found to have significantly lower rates of new-onset dialysis compared with patients undergoing an ECMO BTT strategy. Renal dysfunction is common in patients supported with ECMO^18^ and can be due to hemodynamic alterations associated with cardiogenic shock or secondary to ECMO itself, such as reduction of peripheral organ perfusion^19^ or intravascular hemolysis.^20^ Bridging to a durable VAD before transplantation could give patients additional time for end-organ stabilization before transplantation, thereby minimizing posttransplant complications.

As the first study to investigate outcomes in patients who have undergone a BTB strategy, we show that posttransplant outcomes are acceptable in this population and that BTB candidates may be considered for transplantation. Moving forward, this comparison may be most relevant for patients supported with temporary MCS who do not immediately receive a transplant. According to the 2018 UNOS allocation revision,^2^ patients supported with ECMO for more than 7 days or IABP/tVAD for more than 14 days are downgraded to status 3, making them equivalent in status to those supported with a dischargeable VAD during the 30-day discretionary period. These time points therefore mark an important decision point for whether to transition candidates from temporary to durable MCS. In addition to posttransplant mortality, waitlist mortality will play a vital role in this decision-making process, especially given our data demonstrating significant waitlist mortality for temporary MCS patients. In particular, ECMO candidates in our study had a waitlist mortality of 24% and tVAD candidates had a waitlist mortality of 11%. The additional costs, morbidity, and mortality of prolonged temporary MCS support in an intensive care unit setting must be carefully balanced with the additional waitlist times incurred with transition to a dischargeable VAD. With the significant changes in waitlist criteria following the allocation change, a comparison of waitlist outcomes of BTB and BTT strategy candidates in the previous era, when durable and temporary MCS candidates had equal waitlist status, would not meaningfully reflect waitlist outcomes of the current era. Future areas of investigation should focus on the risks associated with remaining on prolonged temporary MCS support versus transitioning to a dischargeable VAD under the current allocation guidelines.

This study has several limitations. First, this was a retrospective study and was limited by the variables available in the OPTN database. As such, there was limited information on the indications for switching patients from temporary to durable support and other details on the clinical course of these patients. Secondly, the results of this study must be carefully interpreted in the context of a rapidly evolving MCS landscape. Over the past 15 years, there have been technological developments, improvements in devices, and changes in clinical practice that may have influenced posttransplant outcomes. We attempted to adjust for transplant era in 2 ways: by including transplant year and durable LVAD type as covariates in our multivariable models; and by performing a subgroup analysis of patients transplanted during the most recent quartile of our study period (2017-2020). Reassuringly, although sample size was small, our subgroup analysis demonstrated similar short-term mortality. Lastly, this current analysis of posttransplant outcomes in patients undergoing a BTB strategy only accounts for candidates who survive to transplantation and does not include candidates who die on the waitlist. Moving forward, waitlist outcomes will also be important to further explore, as discussed above.

## Conclusions

**Figure 5:**
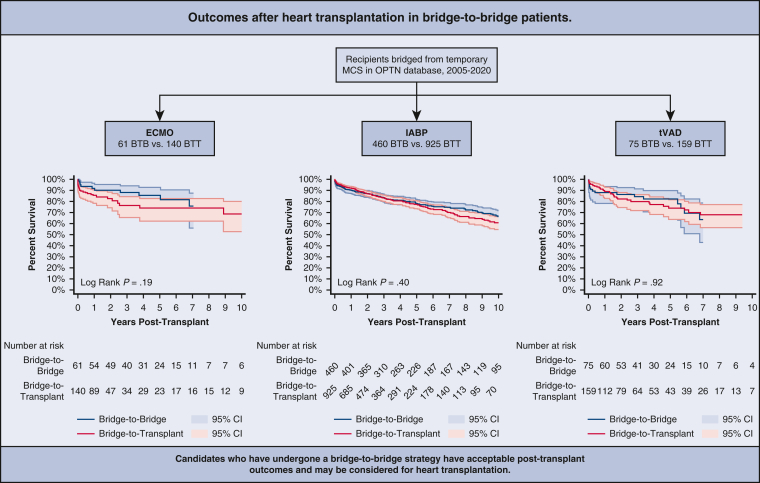
Summary of results of the study. 95% confidence intervals (CI) in shaded area. *MCS*, Mechanical circulatory support; *OPTN*, Organ Procurement and Transplantation Network; *ECMO*, extracorporeal membrane oxygenation; *IABP*, intra-aortic balloon pump; *tVAD*, temporary ventricular assist device; *BTB*, bridge-to-bridge; *BTT*, bridge-to-transplant.

Our study was the first to provide data on the posttransplant survival, both unconditional and conditional, of patients undergoing a BTB strategy. Results from our analysis suggest that posttransplant outcomes were not significantly different between BTB and BTT strategies for patients initially supported with ECMO, IABP, or tVADs. These results suggest that patients who have undergone a BTB strategy before transplantation may be suitable candidates for heart transplantation (Figure 5). As more data following the 2018 allocation change becomes available, future studies should continue to investigate the tradeoff between continued prolonged temporary MCS support and transitioning to durable MCS.

### Conflict of Interest Statement

The authors reported no conflicts of interest.

The *Journal* policy requires editors and reviewers to disclose conflicts of interest and to decline handling or reviewing manuscripts for which they may have a conflict of interest. The editors and reviewers of this article have no conflicts of interest.

## Figures and Tables

**Table E1:** Posttransplant survival analysis of bridge-to-bridge (BTB) versus bridge-to-transplant (BTT) patients from different types of temporary mechanical circulatory support (MCS)

MCS device	Survival time	Bridging strategy	Survival (%)	Hazard ratio (95% CI)	*P* value
ECMO	1 y	BTT	85.5	1∗	
		BTB	91.8	0.54 (0.20-1.43)	.21
	5 y	BTT	73.9	1∗	
		BTB	85.5	0.52 (0.23-1.16)	.11
	5 y conditional (on survival at 1 y)	BTT	86.5	1∗	
		BTB	93.1	0.49 (0.12-1.97)	.31
	10 y	BTT	68.7	1∗	
		BTB	75.8	0.61 (0.29-1.28)	.19
	10 y conditional (on survival at 5 y)	BTT	92.9	1∗	
		BTB	88.6	2.63 (0.23-29.87)	.43
IABP	1 y	BTT	92.2	1∗	
		BTB	90.1	1.28 (0.88-1.85)	.20
	5 y	BTT	77.3	1∗	
		BTB	78.2	0.99 (0.76-1.30)	.96
	5 y conditional (on survival at 1 y)	BTT	83.9	1∗	
		BTB	86.8	0.78 (0.54-1.14)	.20
	10 y	BTT	60.8	1∗	
		BTB	66.3	0.90 (0.71-1.15)	.40
	10 y conditional (on survival at 5 y)	BTT	78.7	1∗	
		BTB	84.9	0.63 (0.37-1.06)	.08
tVAD	1 y	BTT	89.0	1∗	
		BTB	88.0	1.14 (0.51-2.55)	.75
	5 y	BTT	73.9	1∗	
		BTB	82.4	0.76 (0.39-1.49)	.43
	5 y conditional (on survival at 1 y)	BTT	83.1	1∗	
		BTB	93.6	0.36 (0.10-1.28)	.12
	10 y	BTT	68.0	1∗	
		BTB	55.7	1.03 (0.57-1.85)	.92
	10 y conditional (on survival at 5 y)	BTT	91.9	1∗	
		BTB	67.5	**4.39 (1.05-18.38)**	**.04**

Statistical significant (*P* < .05) denoted with boldface type. *CI*, confidence interval; *ECMO*, extracorporeal membrane oxygenation; *IABP*, intra-aortic balloon pump; *tVAD*, temporary ventricular assist device.

∗ Reference category.

**Table E2:** Multivariable 1-year posttransplant survival after adjusting for baseline characteristics

MCS device	Group	Univariate Cox regression		Multivariable Cox regression	
		Hazard ratio (95% CI)	*P* value	Hazard ratio (95% CI)	*P* value
ECMO	BTT	1∗	–	1∗	–
	BTB	0.54 (0.20-1.43)	.21	0.75 (0.05-10.45)	.83
IABP	BTT	1∗	–	1∗	–
	BTB	1.28 (0.88-1.85)	.20	1.32 (0.70-2.49)	.39
tVAD	BTT	1∗	–	1∗	–
	BTB	1.14 (0.51-2.55)	.75	1.17 (0.25-5.60)	.84

*MCS*, Mechanical circulatory support; *CI*, confidence interval; *ECMO*, extracorporeal membrane oxygenation; *BTT*, bridge-to-transplant; *BTB*, bridge-to-bridge; *IABP*, intra-aortic balloon pump; *tVAD*, temporary ventricular assist device.

∗ Reference category.

**Table E3:** Multivariable 10-year posttransplant survival after adjusting for baseline characteristics

MCS device	Group	Univariate Cox regression		Multivariable Cox regression	
		Hazard ratio (95% CI)	*P* value	Hazard ratio (95% CI)	*P* value
ECMO	BTT	1∗	–	1∗	–
	BTB	0.61 (0.29-1.28)	.19	0.38 (0.07-2.04)	.26
IABP	BTT	1∗	–	1∗	–
	BTB	0.90 (0.71-1.15)	.40	0.68 (0.44-1.06)	.08
tVAD	BTT	1∗	–	1∗	–
	BTB	1.03 (0.58-1.85)	.92	1.33 (0.27-6.60)	.73

*MCS*, Mechanical circulatory support; *CI*, confidence interval; *ECMO*, extracorporeal membrane oxygenation; *BTT*, bridge-to-transplant; *BTB*, bridge-to-bridge; *IABP*, intra-aortic balloon pump; *tVAD*, temporary ventricular assist device.

∗ Reference category.

**Table E4:** Subgroup of postoperative outcomes in bridge-to-bridge (BTB) versus bridge-to-transplant (BTT) patients transplanted from 2017 to 2020

MCS device	Postoperative outcome	BTB	BTT	*P* value
ECMO		n = 17	n = 93	
	30-d mortality (%)	100	96.8	.46∗
	1-y mortality (%)	94.1	88.8	.52∗
	Length of stay (d)	20 (16-32)	19 (15-37)	.99
	Acute rejection	5 (29.0)	12 (13.0)	.08
	Treated acute rejection	2 (11.8)	5 (5.4)	.32
	Airway compromise	0 (0.0)	0 (0.0)	
	Stroke	0 (0.0)	9 (10.0)	.35
	Dialysis	1 (6.0)	20 (22.0)	.19
	Pacemaker	0 (0.0)	0 (0.0)	
IABP		n = 85	n = 496	
	30-d mortality (%)	98.8	97.6	.48∗
	1-y mortality (%)	92.8	92.7	.94∗
	Length of stay (d)	16 (12-26)	16 (12-24)	.66
	Acute rejection	29 (34.1)	104 (21.0)	**.008**
	Treated acute rejection	14 (16.5%)	48 (9.7)	.06
	Airway compromise	1 (1.2%)	1 (0.2)	.27
	Stroke	3 (3.5)	15 (3.0)	.80
	Dialysis	13 (15.3)	64 (12.9)	.55
	Pacemaker	0 (0.0)	14 (2.8)	.12
tVAD		n = 24	n = 74	
	30-d mortality (%)	95.8	100	.08∗
	1-y mortality (%)	87.5	93.9	.22∗
	Length of stay (d)	17.5 (14-48)	19 (14-29)	.90
	Acute rejection	6 (25.0)	20 (27.0)	.85
	Treated acute rejection	5 (20.8)	11 (14.9)	.49
	Airway compromise	0 (0)	0 (0)	
	Stroke	2 (8.0)	0 (0.0)	**.01**
	Dialysis	6 (25.0)	10 (14.0)	.19
	Pacemaker	0 (0.0)	1 (1.0)	.57

Values are presented as %, median (interquartile range), or n (%). Statistical significant (*P* < .05) denoted with boldface type. *MCS*, Mechanical circulatory support; *ECMO*, extracorporeal membrane oxygenation; *IABP*, intra-aortic balloon pump; *tVAD*, temporary ventricular assist device.

∗ *P* value for survival data represents log-rank *P* value.
